# Identification of mutation-specific tumor infiltrating lymphocytes from metastatic ovarian cancer

**DOI:** 10.1186/2051-1426-3-S2-P10

**Published:** 2015-11-04

**Authors:** Drew C Deniger, Mini Bharathan, Pasetto Anna, Eric Tran, Jared J Gartner, Todd D Prickett, Paul F Robbins, Steven A Rosenberg

**Affiliations:** 1 NIH/NCI, Bethesda, MD, USA; 2 Surgery Branch/National Cancer Institute / National Institutes of Health, Bethesda, MD, USA; 3 NCI/NIH, Bethesda, MD, USA

## Introduction

Novel interventions are needed for the treatment of women with metastatic ovarian cancer that causes 14,000 deaths each year in the USA. Retrospective analysis of tumor infiltrating lymphocytes (TIL) infused into patients who achieved complete, durable regressions of metastatic melanoma revealed that the responses were likely mediated through recognition of somatic non-synonymous mutations. Furthermore, prospective administration of mutation-specific TIL resulted in an ongoing partial response in a patient with cholangiocarcinoma.

## Methods

We now studied whether TIL obtained from metastatic ovarian cancer recognized tumor mutations. Exome and transcriptome sequencing was performed from resected metastatic ovarian cancer deposits in parallel with growth of TIL fragment cultures in IL-2. Long peptides and tandem minigenes (TMGs) encompassing all mutations were synthesized, introduced into autologous antigen presenting cells, co-cultured with individual TIL fragments and T cell reactivity was determined by interferon-γ ELISPOT and surface expression of 41BB.

## Results

In patient 4046 (280 mutations), 8 of 24 fragments were reactive to TMG15 (86.9% ± 1.5% 41BB^+^ T cells; mean ± SEM). Co-culture with individual long peptides corresponding to the mutations in TMG15 revealed that the T cells recognized the USP9X^Y373C^ mutation, a putative tumor suppressor gene on the X chromosome. Two of 14 fragments from patient 4067 (122 mutations) were responsive to autologous tumor and a cumulative 41BB^+^ expression was observed at 51% and 8% in CD4^+^ and CD8^+^ T cells, respectively, following co-culture with TMG2, TMG5, TMG6 and TMG7. Using UV-exchangeable peptide tetramers, the following candidate minimal epitopes from this patient were identified: ZWILCH^S296F^ from TMG2, SPINT2^H35L^ and TNPO1^M697K^ from TMG5, HIST1H1C^K181R^ from TMG6, and SUN2^P117S^ and HIST1H1C^D72G^ from TMG7. Patient 4097 (332 mutations) had 3 of 24 fragments with recognition of autologous tumor and CD4^+^ T cell reactivity (53%, 74% and 81% 41BB^+^) to mutated HIST1H1B^A71D^ peptide. These fragments had 11% cumulative CD4^+^41BB^+^ T cells when co-cultured with mutated DEF6^L319Q^, GAK^V534M^ and FLOT1^4-11del^ peptides. A fourth fragment had 6% CD4^+^41BB^+^ T cells in response to mutated INPP5K^L176V^ peptide.

## Conclusions

In summary, mutation-specific T cell responses were found in 3 of 4 patients with metastatic ovarian cancer, which opens the opportunity to use these cells for adoptive T cell treatment of advanced ovarian cancer.

